# A secondary bile acid from microbiota metabolism attenuates ileitis and bile acid reduction in subclinical necrotic enteritis in chickens

**DOI:** 10.1186/s40104-020-00441-6

**Published:** 2020-03-13

**Authors:** Mohit Bansal, Ying Fu, Bilal Alrubaye, Mussie Abraha, Ayidh Almansour, Anamika Gupta, Rohana Liyanage, Hong Wang, Billy Hargis, Xiaolun Sun

**Affiliations:** 1 Center of Excellence for Poultry Science, University of Arkansas, 1260 W Maple St. O409, Fayetteville, AR 72701 USA; 2 CEMB, University of Arkansas, Fayetteville, AR 72701 USA; 3 Department of Chemistry, University of Arkansas, Fayetteville, AR 72701 USA

**Keywords:** Bile acid, Chicken, *Clostridium perfringens*, Deoxycholic acid, Intestinal inflammation, Necrotic enteritis

## Abstract

**Background:**

*Clostridium perfringens*-induced chicken necrotic enteritis (NE) is responsible for substantial economic losses worldwide annually. Recently, as a result of antibiotic growth promoter prohibition, the prevalence of NE in chickens has reemerged. This study was aimed to reduce NE through titrating dietary deoxycholic acid (DCA) as an effective antimicrobial alternative.

**Materials and methods:**

Day-old broiler chicks were assigned to six groups and fed diets supplemented with 0 (basal diet), 0.8, 1.0 and 1.5 g/kg (on top of basal diet) DCA. The birds were challenged with *Eimeria maxima* (20,000 oocysts/bird) at d 18 and *C. perfringens* (10^9^ CFU/bird per day) at d 23, 24, and 25 to induce NE. The birds were sacrificed at d 26 when ileal tissue and digesta were collected for analyzing histopathology, mRNA accumulation and *C. perfringens* colonization by real-time PCR, targeted metabolomics of bile acids, fluorescence *in situ* hybridization (FISH), or terminal deoxynucleotidyl transferase dUTP nick end labeling (TUNEL) assay.

**Results:**

At the cellular level, birds infected with *E. maxima* and *C. perfringens* developed subclinical NE and showed shortening villi, crypt hyperplasia and immune cell infiltration in ileum. Dietary DCA alleviated the NE-induced ileal inflammation in a dose-dependent manner compared to NE control birds. Consistent with the increased histopathological scores, subclinical NE birds suffered body weight gain reduction compared to the uninfected birds, an effect attenuated with increased doses of dietary DCA. At the molecular level, the highest dose of DCA at 1.5 g/kg reduced *C. perfringens* luminal colonization compared to NE birds using PCR and FISH. Furthermore, the dietary DCA reduced subclinical NE-induced intestinal inflammatory gene expression and cell apoptosis using PCR and TUNEL assays. Upon further examining ileal bile acid pool through targeted metabolomics, subclinical NE reduced the total bile acid level in ileal digesta compared to uninfected birds. Notably, dietary DCA increased total bile acid and DCA levels in a dose-dependent manner compared to NE birds.

**Conclusion:**

These results indicate that DCA attenuates NE-induced intestinal inflammation and bile acid reduction and could be an effective antimicrobial alternative against the intestinal disease.

## Background

NE is an intestinal disease in chickens. The main causative pathogen of NE is a spore-forming, anaerobic, and gram-positive bacterium *Clostridium perfringens* and NE is often associated with the predisposing factor of *Eimeria maxima* and/or *E. acervulina* infection. Birds suffering acute NE show sudden death of up to 50% mortality [1]; however, the more common form of NE is subclinical with neither obvious clinical signs present nor peak mortality. Antimicrobials have been used to prevent coccidiosis and NE [2], but the growing concerns on antimicrobial resistance promotes the restriction of their usage [2, 3].

The practice to withdraw antimicrobials in poultry production is facing a number of challenges. Among them is the elevated productivity loss from chicken diseases, such as NE. Recently, the reemergence of NE has costed poultry industry $6 billion annually worldwide [4]. Severely ill and deceased NE birds show fragile intestines ballooned with gas and a foul-smelling brown fluid [5]. The severity of NE is often diagnosed using gross lesion scores [6]. At the cellular level, the intestinal tract of acute NE birds displays severe small intestinal inflammation from an infiltration of massive immune cells into the lamina propria, villus epithelial line necrosis and crypt hyperplasia [7–9]. Progress has been made in understanding the risk factors influencing the outcome of NE such as *C. perfringens* virulence, coccidiosis and feed [10]. However, little progress has been made in developing strategies for antibiotic alternatives to prevent and treat NE.

*C. perfringens* colonizes the intestinal tract among normal chicken microbiota. The presence of high doses of *C. perfringens* in the intestine does not sufficiently induce NE and the count alone does not associate with NE [11–13]; therefore, the development of NE is dependent on *C. perfringens* toxins and predisposing factors, such as coccidiosis, feed and microbiota. Coccidiosis is the most comment predisposing factors in field NE cases [14]. It is well known that different strains of *C. perfringens* produce a variety of toxins such as alpha (CPA, type A), beta toxin 2 (CPB2), enterotoxin (CPE), necrotic enteritis B-like toxin (NetB), TpeL and others [15]. Among them, *C. perfringens* isolated from NE birds are mainly type A strains. Researchers in Australia have reported that NetB induced NE in chickens, but not CPA [16]. However, *C. perfringens* isolated from clinical NE birds isn’t always associated with virulent genes of *netB*, *cpb2*, *tpeL* or *cpe* [17, 18]. Thus, more researches are needed to investigate the relationship between NE and *C. perfringens* virulent factors, particularly in the presence of predisposing factor of coccidiosis.

Human and animal intestines harbor up to trillions of microbes which regulate various host functions such as the intestinal barrier, nutrition and immune homeostasis [19–22]. Microbiota composition is altered in NE birds compared to uninfected birds [23], which may lead to bile acid changes in the host. At the gut level, microbiota reconstitution by transplantation has shown tremendous success against recurrent *Clostridium difficile* infection [24]. The reduction of *C. difficile* infection is attributed to the secondary bile acids produced by the transplanted normal microbiota member *C. scindens* [25]. Bile acids are synthesized and conjugated in the liver, including tauro- or glycol-cholic acids (TCA or GCA) and tauro- or glycol-chenodeoxycholic acids (TCDCA or GCDCA). After the conjugated bile acids are released, they are metabolized in the intestine by various gut microbiota through deconjugation (e.g. CA or CDCA) and transformed into secondary bile acids, such as deoxycholic acid (DCA), lithocholic acid (LCA) or ursodeoxycholic acid (UDCA) [26]. The majority of bile acids (> 95%) are effectively absorbed in the intestine through enterohepatic circulation/cycle [26]. Bile acids have been reported in association with a variety of chronic diseases [27, 28], but new evidence sheds light on the beneficial property of secondary bile acids in health and disease, such as increasing gut motility [29] and reducing *C. difficile* infection [25]. It remains largely unknown how dietary bile acids modulate intestinal bile acid pool in health and disease.

Anaerobic microbiota and its metabolite DCA prevent *Campylobacter jejuni*-induced intestinal inflammation [30] in mice and bacterial colonization in chickens [31]. Recently, we found that *E. maxima* and *C. perfringens* induced acute NE, body weight loss and intestinal inflammation, which was effectively attenuated by dietary DCA at 1.5 g/kg [9]. We reasoned that dietary DCA would modulate NE in a dose response manner. To address this possibility, we conducted a following up chicken experiment. In the current experiment, different dietary doses of DCA were used to prevent subclinical NE and to investigate the impact of dietary DCA on intestinal bile acid pool. The results from this study will help the understanding of NE pathogenesis and assist in developing new antimicrobial alternatives against NE.

## Materials and methods

### Chicken experiment

The experiment was conducted at the Poultry Health Laboratory of University of Arkansas at Fayetteville. All the experimental procedures were approved by the Animal Care and Use Committee of the University of Arkansas. A total of 96 male, 1-day-old broiler chicks were obtained from Cobb-Vantress (Siloam Springs, AR). Upon arrival, broiler chicks were neck-tagged, individually weighed and randomly assigned to one of 6 floor pens in a controlled age appropriate environment. Birds were fed with 0, 0.8, 1.0 and 1.5 g/kg DCA (Alfa Aesar, MA, US) on top of basal diets from d 0 to 26. The basal diets were a corn-soybean meal-based starter diet during d 0 to10 and a grower diet during d 11 to 26 [9]. Birds were infected with 20,000 sporulated *E. maxima* M6 oocysts at d 18 [9]. *E. maxima* used was propagated and sporulated 1 yr before the chicken experiment. The birds were then infected with *C. perfringens* [32, 33] with 10^9^ CFU/bird at d 23, 24 and 25 [9]. This *C. perfringens* isolate was confirmed to be *netB* and *cpe* positive by PCR. Chicken body weight was measured at d 0, 18, 23 and 26. Birds were sacrificed at d 26 to collect ileal tissue and digesta for analysis of histopathology, bacterial colonization and inflammation. In a separate trial, 16 birds/pen were challenged as before and feed intake was calculated.

### Histopathology analysis of intestinal inflammation

Distal jejunal (2 cm) and proximal ileal tissue (8 cm) adjacent to Meckel’s diverticulum was removed, Swiss-rolled, and fixed in 10% phosphate-buffed formalin (pH 7.4) overnight at 4 °C. Tissue samples were then embedded in paraffin and sections (5 μm) were cut, processed and stained with H&E at the Histology Laboratory in the Department of Poultry Science at the University of Arkansas in Fayetteville, Arkansas. Each Swiss-roll slide at ileum was evaluated for ileal inflammation based on the four-characteristic NE lesion score system. The four characteristics of NE lesion are level of immune cell infiltration in lamina propria, villus length, crypt hyperplasia and tissue ulceration [9]. Briefly, continuous (decimal) scoring method was used on scale of 0-4 where 0 indicates: no inflammation, villi and crypt intact; score 1: small number infiltration cells in laminar propria of villi and crypts or villi minimally shortened; score 2: more extensive infiltration cells in laminar propria of villi and crypts, villi shortened > 1/4 and edema, or crypt hyperplasia; score 3: pronounced infiltration cells in laminar propria of villi, crypts, submucosa, and muscularis, villi shortened > 1/2 and edema, or crypt hyperplasia and regeneration; and score 4: necrosis, villus diffuse, ulcers, crypt abscesses, or transmural inflammation (may extend to serosa). Images of representative histopathology were acquired using a Nikon TS2 fluorescent microscope.

### *C. perfringens* colonization in ileal lumen using real-time PCR and fluorescence *in situ* hybridization (FISH)

Ileal digesta samples were collected from birds scarified on d 26. Total DNA was extracted using phenol/chloroform method as previously described [9]. Real-time PCR was performed to quantify the colonization level of *C. perfringens* in ileal digesta targeting 16S rDNA and using a bacterial standard as described before [9]. FISH was performed to visualize *C. perfringens* intestinal colonization using histology slides as described before [9].

### Host inflammatory response and bacterial gene using real-time PCR and terminal deoxynucleotidyl transferase dUTP nick end labeling (TUNEL) assay

The host immune response of inflammatory gene expression was evaluated in ileal tissue samples. The total RNA was extracted from ileal tissue samples of birds sacrificed at d 26 using TRIzol as described before [9, 34] and cDNA was prepared using M-MLV (NE Biolab). The accumulation of proinflammatory genes of *Infγ*, *Mmp9*, *Il17a, Il22* and *Il23* in ileum tissue were determined using SYBR Green PCR Master mix (Bio-Rad) on a Bio-Rad 384-well Real-Time PCR System. The primer sequences of each gene were described before [9] and here: *Il22*_forward: 5′-CTGCCCATAGCTGCAGTACA-3′; *Il22*_reverse: 5′- 3′; *Il23_*forward: 5′-ATGCATTGCGATGTCTGAAG-3′; *Il23*_reverse: 5′-ACTTGGGTGCTTCCAAGATG-3′. Gene expression of fold changes in uninfected birds was calculated using ΔΔCt method [35] and *Gapdh* was used as the internal control.

*C. perfringens* virulent DNA genes and 16S (internal control) were detected using real-time PCR and the primer sequences were: *netB*_forward: GGAAAAATGAAATGGCCTGA; *netB*_reverse: GCACCAGCAGTTTTTCCTTC; *cpe*_forward: CAACTGCTGGTCCAAATGAA; *cpe*_reverse: GCATCTTTCGCCAGTTTCAA; 16S_forward: AGGAGCAATCCGCTATGAGA; 16S_reverse: GTGCAATATTCCCCACTGCT.

### Quantification of ileal bile acids using targeted metabolomics

The ileal digesta was collected and the bile acid composition was analyzed using multiple reaction monitoring (MRM) mass spectrometry in Statewide Mass Spectrometry Facility at the University of Arkansas in Fayetteville, Arkansas. Five or six ileal digesta samples from each group were used for extraction of bile acids. The Intestinal bile acids were extracted using chloroform/methanol. To identify and quantify bile acids, MRM methods have been developed for tauro- and glyco-cholic acid (TCA/GCA), tauro- and glyco-chenodeoxycholic acid (TCDCA/GCDCA), cholic acid (CA), deoxycholic acid (DCA), lithocholic acid (LCA), ursodeoxycholic acid (UDCA) isotopically labeled (some were with deuteriums and some were with five deuteriums) and unlabeled standards. The methods were fully optimized using data acquisition software attached to Shimadzu UPLC-20A/LC-30A coupled with Shimadzu 8050 triple quadrupole mass spectrometer at their respective retention times. Once the methods were optimized, the samples with isotopically labeled internal standards were analyzed along with standards of varying concentrations. Calibration curves with internal standards were used to estimate bile acid concentration. The use of the internal standard accounts for the sample loss during the extraction procedure. LC-MS/MS analysis was performed in the negative-ion mode. Methodology is similar to what has been described before [36]. LC separation was performed using a C18 column. The flow rate was 0.3 mL/min and sample volumes of 1 μL were injected. The three most intense multiple reaction monitoring (MRM) fragments for each bile acid standard were fully optimized at their respective retention time window using Shimadzu Labsolution software as described before. From the three MRM channels, the MRM event corresponding to the most intense fragment ion was used as the “quant” ion, and the other two fragment ions were used as reference ions for identification purposes. Reference ion ratios were calculated compared to the “quant” ion, and unknown samples were required to meet within 30% of the reference ion ratio of the standard.

### Statistical analysis

Differences between treatments were analyzed using One-way ANOVA followed by Dunnette’s multiple comparison test using Prism 7.0 software. Data were also analyzed using the nonparametric Mann-Whitney *U* test using Prism 7.0 software. Values are shown as a mean of samples in the treatment ± standard error of the mean as indicated. Experiments were considered statistically significant if *P* values were < 0.05. Regression models were constructed to generate the correlations between dietary DCA levels and host responses (body weight gain, histopathological score and bile acids). R^2^ was used to indicate the strength of correlation.

## Results

### DCA attenuated subclinical NE-induced ileal inflammation

**Fig. 1:**
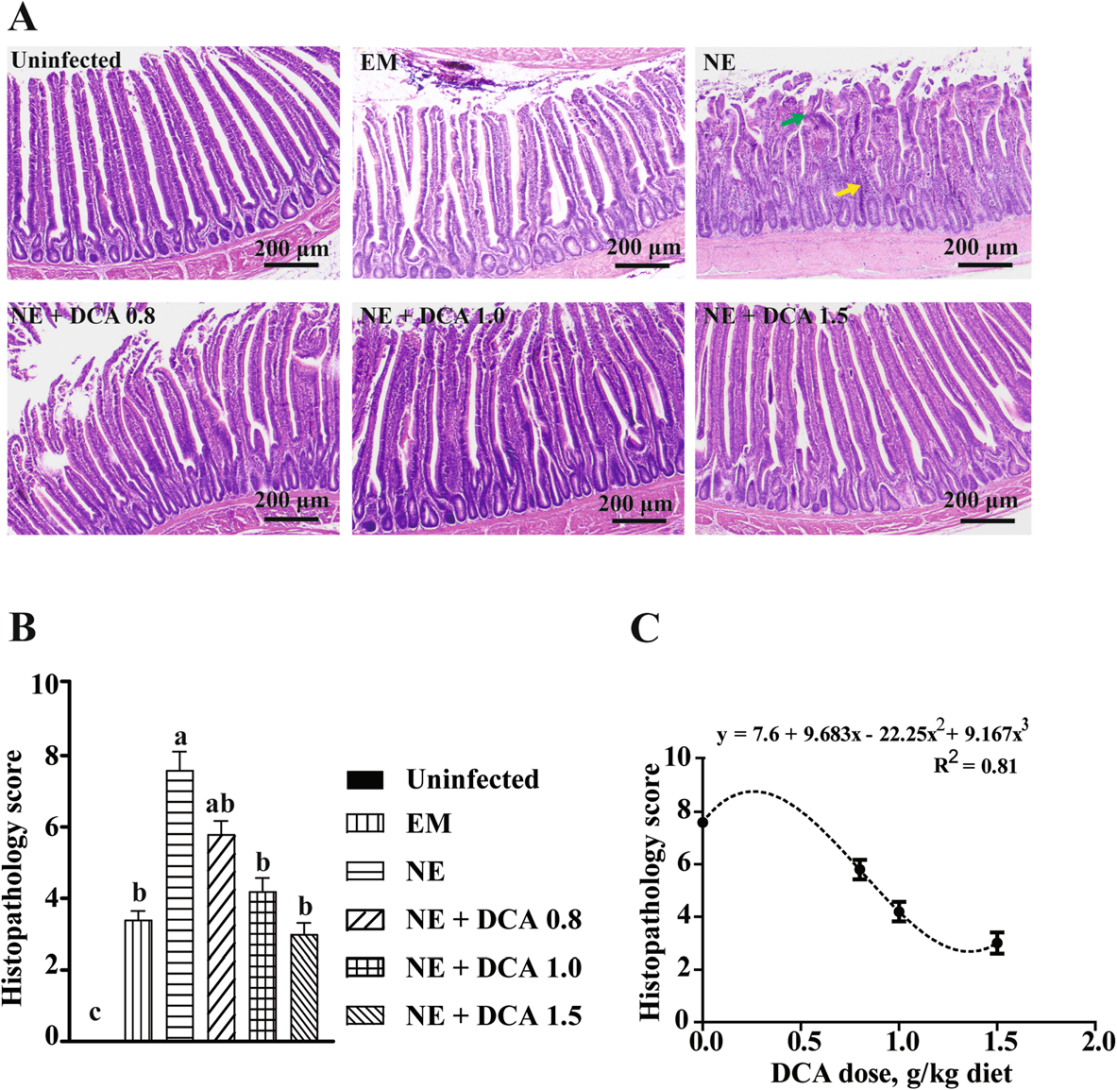
DCA prevented subclinical NE-induced ileal inflammation. Cohorts of 16 birds were fed diets supplemented with 0, 0.8, 1.0 and 1.5 g/kg DCA. Birds were challenged with *E. maxima* at d 18 and *C. perfringens* at d 23, 24, and 25. Randomly selected five birds from each treatment groups were sacrificed and ileal tissue samples were collected. **a** Representative images of H&E-stained ileal tissue sections showing immune cell infiltration (yellow arrow) and blunted villi (green arrow). **b** Histopathology score of ileal tissue. **c** Fitting curve for histopathology scores in response to different doses of DCA. All graphs showed mean ± SEM. Different letters of a, b and c mean *P* < 0.05. Results are representative of 3 independent experiments. Scale bar is 200 μm

In contrast to the previous experiment, NE birds in current experiment showed mild diarrhea (wet bedding) and no mortality. We then examined NE disease development at the cellular level using histopathology analysis. Consistent with the mild subclinical signs, ileum in NE birds showed moderate ileitis with mild crypt hyperplasia, villus shortening and infiltration of immune cells in lamina propria (Fig. 1a). Significantly, dietary DCA at 0.8, 1.0, and 1.5 g/kg reduced the intestinal inflammation and histopathological scores by 23%, 48% and 61% respectively compared to NE birds (Fig. 1a and b). To develop a deeper understanding about the relationship between dietary DCA levels and NE ileitis, a dose response curve was generated. Notably, the lowest dose of DCA to attenuate subclinical NE was at 0.5682 g/kg (Fig. 1c), while DCA at 1.359 g/kg would reduce subclinical NE to the lowest score of 2.67.

**Fig. 2:**
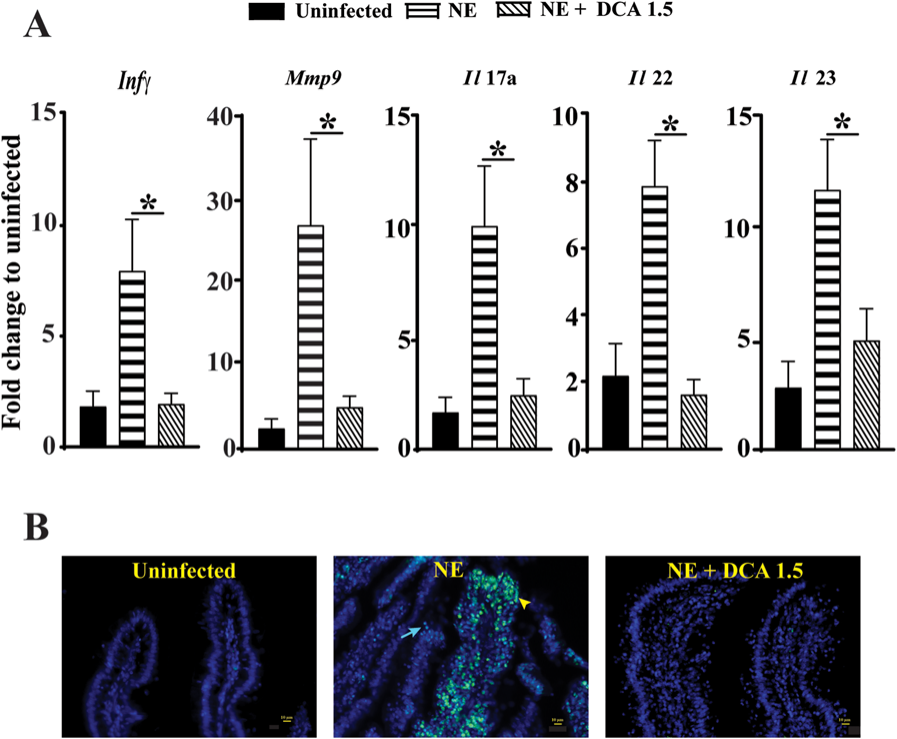
DCA reduced NE-induced host inflammatory response. Cohorts of broiler chickens were fed with different doses of DCA and infected as in Fig. 1. **a** Ileal *Infγ*, *Mmp9*, *Il*17A, *Il*22 and *Il*23 mRNA fold changes relative to uninfected birds and normalized to *Gapdh.***b** Representative TUNNEL assay images showing epithelial (Arrow) and immune cell cell apoptosis (arrow head). Scale bar is 10 μm. All graphs depict mean ± SEM. * indicates *P* < 0.05. Results are representative of 3 independent experiments

Because DCA at 1.5 g/kg exhibited best potential to reduce NE, we focused our attention on DCA at that dose against host inflammatory mediator mRNA expression using real-time PCR. NE induced significantly higher accumulation of ileal inflammatory mRNA mediators of *Infγ*, *Mmp9*, *Il17a, Il22*, and *Il23* by 3.32, 10.6, 6.02, 3.56 and 4.16 folds, respectively, compared to uninfected birds (Fig. 2a). Remarkably, DCA at 1.5 g/kg attenuated the NE-induced inflammatory mediators of *Infγ, Mmp9*, *Il-17a, Il-22*, and *Il-23* by 76%, 81%, 76%, 79%, and 58%, respectively. To further understand how DCA against subclinical NE, TUNEL assay was performed to examine cell death. Consistent with the inflammatory mediator gene expression, NE induced substantial epithelial (arrow) and lamina propria immune cell death (arrow head) in ileal villi of the subclinical NE birds (Fig. 2b), whereas dietary DCA at 1.5 g/kg attenuated the effect.

### DCA reduced subclinical NE-induced body weight gain loss

**Fig. 3:**
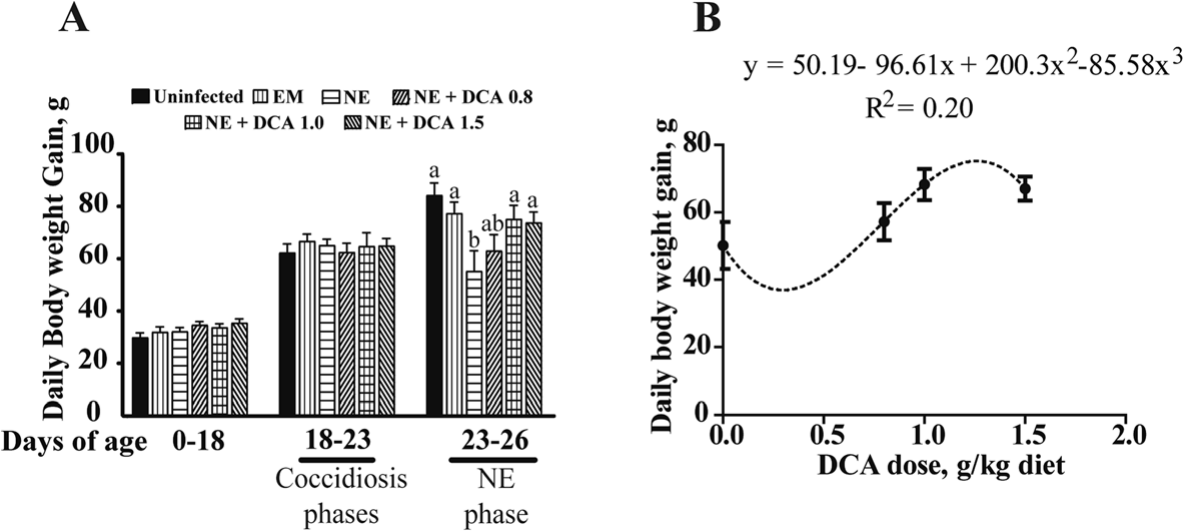
DCA prevented NE induced body weight gain loss. Cohorts of 16 broiler chicks were fed basal and DCA supplemented diets (0.8, 1.0 and 1.5 g/kg) and infected as in Fig. 1. **a** Bird body weight gain was measured at d 18, 23 and 26. Showed were periodic body weight gain at d 0 to 18, 18 to 23 and 23 to 26. **b** Fitting curve for daily body weight gain at d 23 to 26 in response to different doses of DCA. All graphs showed mean ± SEM. Different letters of a and b mean *P* < 0.05. Results are representative of 3 independent experiments

We then evaluated how subclinical NE impacted birds’ daily body weight gain. Birds grew comparably between different groups during the uninfected phase of d 0 to 18 (Fig. 3a). Interestingly, there was a minimal difference of body weight gain between uninfected and *E. maxima*-infected birds during *E. maxima* phase (EM) from d 18 to 23, suggesting a mild coccidiosis from the *E. maxima* infection in this experiment. Notably, body weight gain in NE birds was reduced by 28% compared to uninfected birds during NE phase from d 23 to 26 (155 vs. 110.7 g/d, *P* < 0.05); an effect attenuated by birds fed with 1.0 and 1.5 g/kg DCA diet (110.7 vs. 150 and 148 g/d, *P* < 0.05). To have further understanding of the relationship between dietary DCA levels and body weight gain in NE, a dose response curve was generated. The lowest dose of DCA to increase body weight gain compared to NE was 0.68 g/kg (Fig. 3b), while DCA at 1.262 g/kg would increase body weight gain by 75.266 g/d.

### DCA reduced *C. perfringens* colonization in ileum

**Fig. 4:**
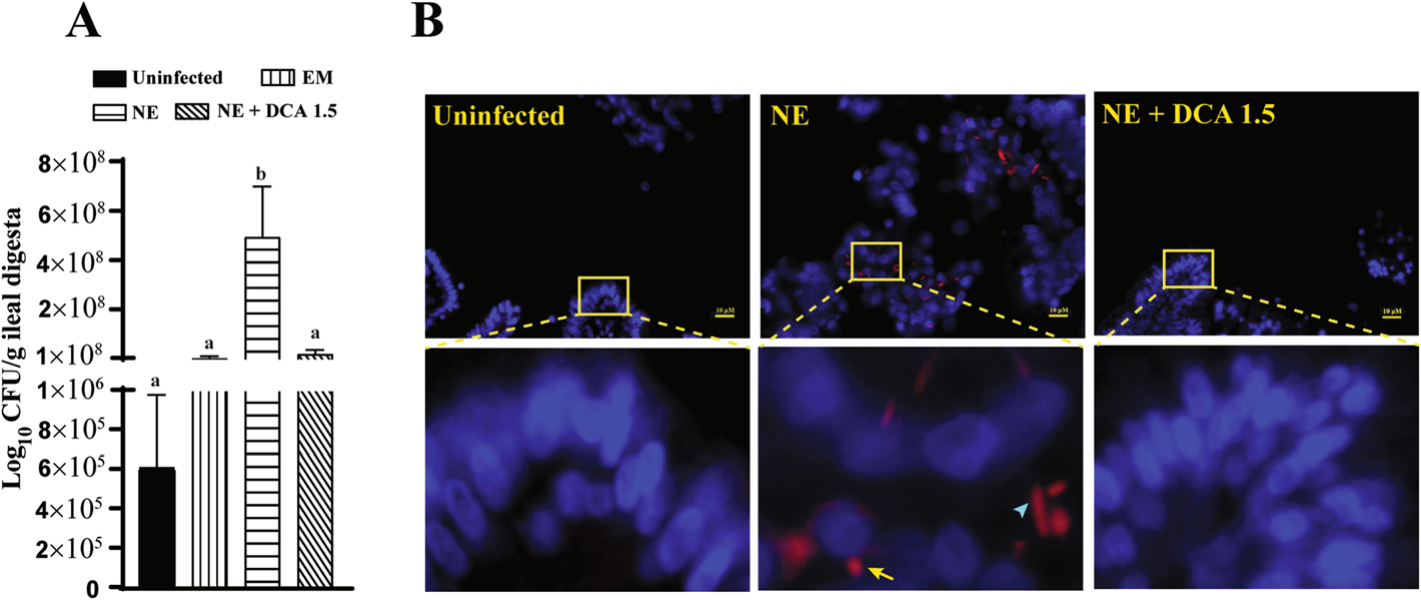
DCA reduced *C. perfringens* ileal colonization. Cohorts of broiler chickens were fed with different doses of DCA and infected as in Fig. 1. **a***C. perfringens* was quantified in ileal digesta using PCR. **b***C. perfringens* was visualized in ileal tissue using FISH. All graphs showed mean ± SEM. Different letters of a and b mean *P* < 0.05. Results are representative of 3 independent experiments. Scale bar is 10 μm

Next, we quantified *C. perfringens* colonization using real-time PCR. Notably, *C. perfringens* in ileum colonized more than 2 logs in NE birds compared to uninfected birds (Fig. 4a). Apart from a previous report of DCA against acute NE [9], DCA at 1.5 g/kg reduced *C. perfringens* colonization by more than 5 folds compared to NE birds. To reveal the spatial distribution of *C. perfringens*, FISH was also performed on the histology tissue slides to reveal the ileal distribution of *C. perfringens*. Interestingly, *C. perfringens* in uninfected birds was barely detected in the lumen and tissue of ileum (Fig. 4b), although it was detected in PCR assay. *C. perfringens* in rod- (arrow head) or spore-shape (arrow) was present in ileal lumen and inside of the villi of subclinical NE birds. *C. perfringens* was barely visualized in birds fed DCA at 1.5 g/kg.

### Dietary DCA restored NE-induced total bile acid level reduction

**Fig. 5:**
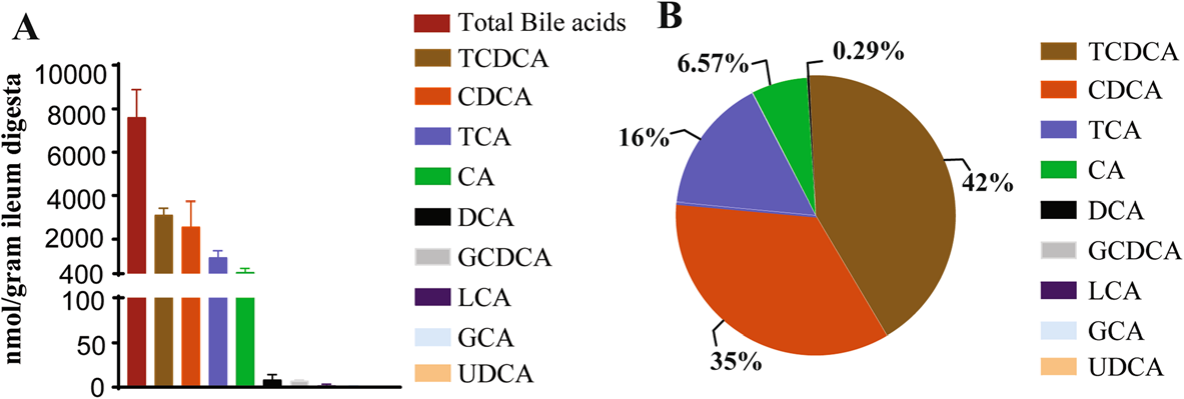
Composition of bile acids in ileal digesta of healthy birds. Bile acids in ileal digesta of 5 healthy birds were extracted and quantified using targeted metabolomics. **a** Bile acids quantification in the healthy bird ileum. **b** Relative composition of bile acid in the healthy birds. All graphs showed mean ± SEM. Results are representative of 3 independent experiments

Bile acids in intestinal digesta were quantified using targeted metabolomics. Notably, the level of total bile acids was at 7,638 nmol/g ileal digesta of uninfected birds (Fig. 5a). Proportionally, the bile acid pool was comprised of 58% conjugated (TCDCA, GCDCA, TCA and GCA) and 42% deconjugated bile acids (CDCA, CA, DCA, LCA and UDCA) (Fig. 5b). Conjugated and deconjugated primary bile acids (TCDCA, GCDCA, TCA, GCA, CDCA and CA) constituted 99.6% of total bile acids in ileum, whereas secondary bile acids of DCA, LCA and UDCA comprised only 0.4% of total bile acids.

**Fig. 6:**
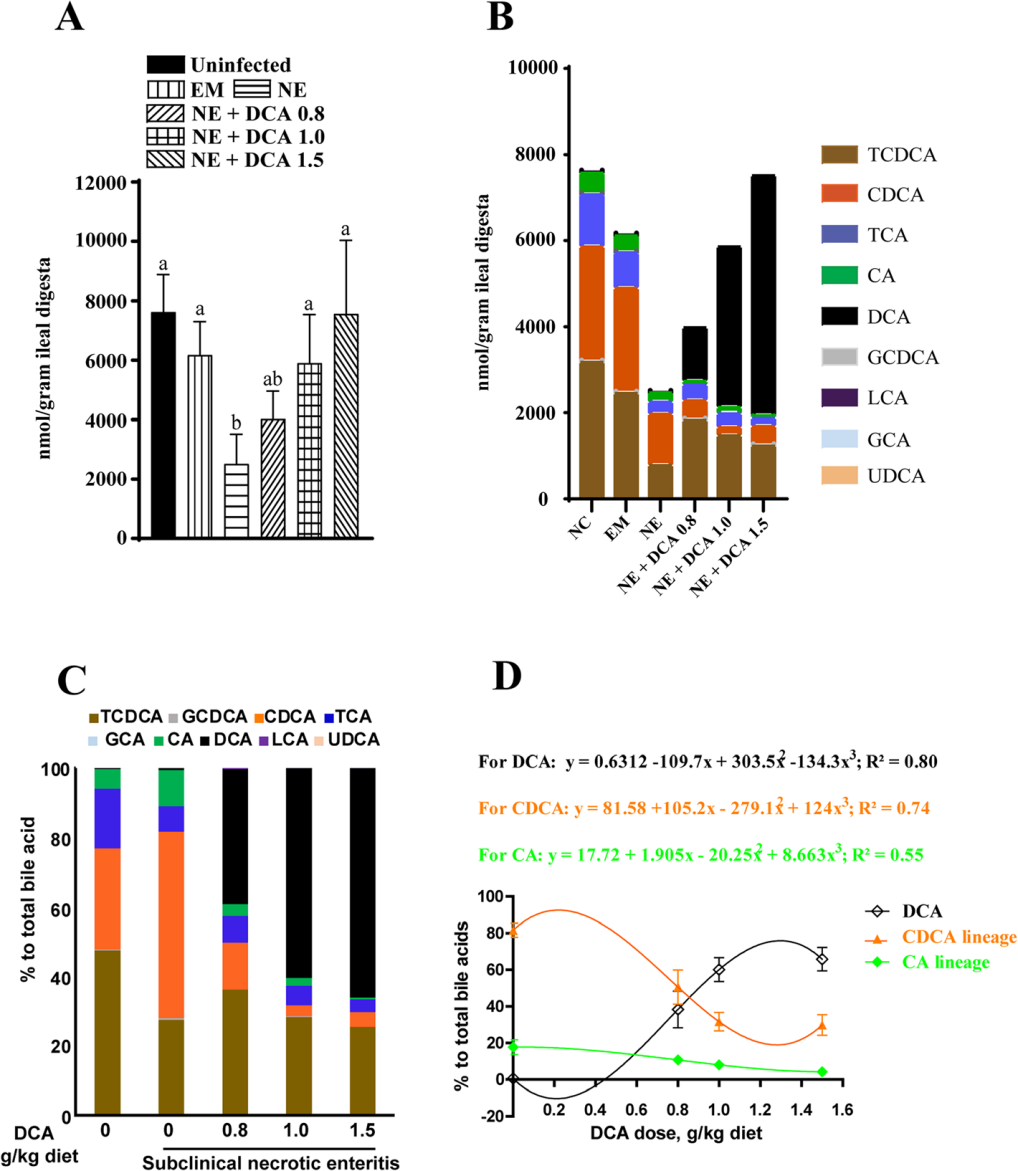
DCA modulated NE-induced bile acid reduction. Cohorts of 16 broiler chicks were fed basal and DCA supplemented diets (0.8, 1.0 and 1.5 g/kg) and infected as in Fig. 1. **a** Total bile acids quantification in NE and DCA treated birds. **b** Individual bile acids quantification in the birds. **c** Relative composition of bile acid in the birds. **d** Fitting curve for DCA, CDCA lineage (T/GCDCA, CDCA), and CA lineage (T/GCA, CA) in response to different doses of DCA. All graphs showed mean ± SEM. Results are representative of 3 independent experiments

We then examined the impact of NE and dietary DCA on ileal bile acid pool. NE reduced the level of ileal total bile acids by 67% compared to uninfected birds (7,638 vs. 2,530 nmol/g), whereas *E. maxima* infection did not significantly reduce the bile acid level (Fig. 6a). Meanwhile, subclinical NE infection reduced feed intake during NE phase only by 14% (Supple Fig. 1). Dietary DCA at 0.8, 1.0, and 1.5 g/kg increased the total bile acid levels in a dose dependent manner (4,000, 5,900, and 7,500 nmol/g, respectively) (Fig. 6b). As a result, the relative proportion of DCA in ileal digesta increased from 0.20% in NE birds to 31%, 63%, 74%, respectively, in birds fed with a DCA diet at 0.8, 1.0, and 1.5 g/kg (Fig. 6c). To further understand the relationship between dietary DCA supplementation and ileal bile acid composition, we drew three correlation lines (Fig. 6d). From the figure, it was possible to predict ileal bile acid composition based on dietary bile acid supplementation, or vice versa.

## Discussion

In current study, the birds experienced subclinical NE possibly because of mild coccidiosis from infection with old *E. maxima*. Importantly, DCA prevented the subclinical NE on intestinal inflammation and body weight gain loss in a dose dependent manner. Dietary DCA increased total bile acid level and modulated bile acid composition in ileum. The results from this study indicate that dietary DCA is able to reduce subclinical NE and modulate intestinal bile acid reduction.

Many predisposition factors shape the outbreak and severity of NE, such as coccidiosis, feed and *C. perfringens* virulence [10]. Specifically, *E. maxima* invades and induces lesions in jejunum and ileum [37], where the NE lesions are often observed [14]. The spatial overlap between NE- and *E. maxima*-induced lesions implicate coccidiosis as the most important predisposed factor for the subsequent NE. Consistent with this notion, NE birds in this study experienced mild body weight gain loss during the NE phase after a mild EM phase on body weight gain. This result highlights the important influence of coccidiosis on the outcome of NE. DCA was able to prevent the body weight gain loss in a dose dependent manner, following the construction of the 3^rd^ order polynomial fitting model using dietary DCA levels and body weight gain during NE phase in Fig. 3b. Interestingly, DCA at 1.5 g/kg was not the optimal dose against NE on body weight gain loss. The lowest DCA dose against subclinical NE-induced body weight gain loss was 0.68 g/kg feed, suggesting that a DCA dose lower than 0.68 g/kg feed would result in less body weight gain compared to subclinical NE birds. The optimal DCA against subclinical NE on body weight would be 1.262 g/kg feed. Future work is needed to dissect which factors contribute to the minimal and optimal doses of DCA against subclinical NE.

The intestinal tract of NE birds often showed a ballooned shape filled with a rotten egg smelling gas, which is the result of an overgrowth of *C. perfringens* [5]. DCA as low as 50 nmol/L inhibits *C. perfringensin vitro* growth in tryptic soy broth [9]. Consistently, DCA reduced *C. perfringens* intestinal luminal colonization levels in this subclinical NE. This result is different from our previous DCA against acute NE [9], where DCA doesn’t significantly reduce *C. perfringens* luminal colonization. Bile acids/salts are often reported to be broad antimicrobial agents [38]. Recent advancement indicates that specific bile acids inhibit respective bacterial growth. For example, secondary bile acid DCA, but not primary bile acid CA, reduces *C. difficilein vitro* growth [25]. Interestingly, despite the presence of *C. perfringens* in all groups of birds by PCR assay, *C. perfringens* was surprisingly barely detected in both uninfected and NE birds fed 1.5 g/kg DCA using FISH. The different results between FISH and PCR may be related to the nonspecific DNA amplification by PCR assay, whereas the FISH assay is more specific and reliable. Notably, rod- and spore-shape *C. perfringens* were easily visualized in ileal lumen and tissue which suggests that *C. perfringens* might experience sporulation and produce enterotoxin (CPE) during this subclinical NE. CPE is implicated in food borne diseases [15] but is rarely investigated in chicken NE. Future work on revealing the role of CPE on NE is greatly needed.

The hallmark of acute NE at cellular level is the severe small intestinal inflammation and necrosis in lower jejunum and upper ileum of chickens [9]. Intestinal tissue in the subclinical NE birds of this experiment showed less extended inflammation with shortening villi, crypt hyperplasia and immune cell infiltration. The intestinal inflammation was consistent with an increased expression of proinflammatory mediators of *Infγ, Mmp9*, *Il-17a, Il-22*, and *Il-23*. DCA was able to prevent the increased inflammatory mediators. Consistent with the previous report of DCA against acute NE on intestinal inflammation [9], a high dose of DCA at 1.5 g/kg feed attenuated NE-induced intestinal inflammation. Furthermore, DCA attenuated intestinal inflammation of histopathology in a dose dependent manner after the construction of the 3rd order polynomial fitting model using dietary DCA levels and an intestinal histopathological score in Fig. 1c. The lowest DCA to reduce a subclinical NE score was at 0.5682 g/kg, while DCA at 1.359 g/kg was the optimal dose to attenuate the NE. Further work is needed to understand the relationship between DCA doses and NE-induced intestinal inflammation.

Finally, subclinical NE birds with mild diarrhea apparently experienced 67% less total bile acids in ileal content compared to uninfected birds, whereas NE birds had 28% less body weight gain loss compared to uninfected birds. The inflammatory bowel disease patients, especially those affecting the distal ileum, often show bile acid malabsorption [39, 40]. Interestingly, the expression of the apical sodium/BA cotransporting polypeptide responsible for ileal BA reabsorption is reduced in ileal biopsies from patients with Crohn’s disease free of any signs of inflammation [41]. Although the birds in this experiment had a subclinical NE with no obvious diarrhea present, the loss of bile acids in excrete from enterohepatic cycle in the birds might be one of the factors. In the experiment, dietary DCA was able to compensate the loss of total bile acid reduction in association with reduced NE on body weight gain loss, intestinal histopathology and inflammation. Bile acid synthesis in liver is regulated by the enterohepatic circulation, but how the impact of individual bile acid on bile acid pool is largely unclear [42]. Cholate is an important negative regulator of bile acid synthesis through the nuclear receptor farnesoid X receptor and sterol 12α-hydroxylase gene (*Cyp8b1*) in mice [43]. It remains unclear whether the protection from dietary DCA comes from actions to specifically antagonize *C. perfringens* or just to restore total intestinal bile acid pool. Following up research is much needed to clarify the underlying mechanism.

## Conclusion

The data presented in this study reveals that the microbial metabolic product secondary bile acid DCA attenuates NE by reducing *C. perfringens* colonization, host inflammatory response and bile acid reduction in a dose-dependent manner. These findings highlight the importance of revealing the molecular relationship between infectious pathogen, bile acids and NE resistance/susceptibility. Discoveries presented here could be applied to control NE and other intestinal diseases targeting microbiome and host response.

## Supplementary information

**Additional file 1: Supplement Figure 1.** Subclinical necrotic enteritis infection reduces feed intake during NE phase. Feed intake of d 23–26 was compared among uninfected, *E. maxima* and NE groups. The graph showed mean ± SEM. Results are representative of 3 independent experiments.

## Data Availability

Not applicable.

## Abbreviations

BW: Body weight
CA: Cholic acid
CDCA: Chenodeoxycholic acids
CFU: Colony forming unit
DCA: Deoxycholic acid
FISH: Fluorescence *in situ* hybridization
GCA: Glycol-cholic acids
GCDCA: Glycol-chenodeoxycholic acids
LCA: Lithocholic acid
NE: Necrotic enteritis
TCA: Tauro-cholic acids
TCDCA: Tauro-chenodeoxycholic acids
TSB: Tryptic Soy Broth
TUNEL: Terminal deoxynucleotidyl transferase dUTP nick end labeling assay
UDCA: Ursodeoxycholic acid
