# Supplementary figures and images for: A secondary bile acid from microbiota metabolism attenuates ileitis and bile acid reduction in subclinical necrotic enteritis in chickens
Source: J Anim Sci Biotechnol. 2020 Mar 13;11:37. doi: 10.1186/s40104-020-00441-6 (PMC7069026; doi:10.1186/s40104-020-00441-6)

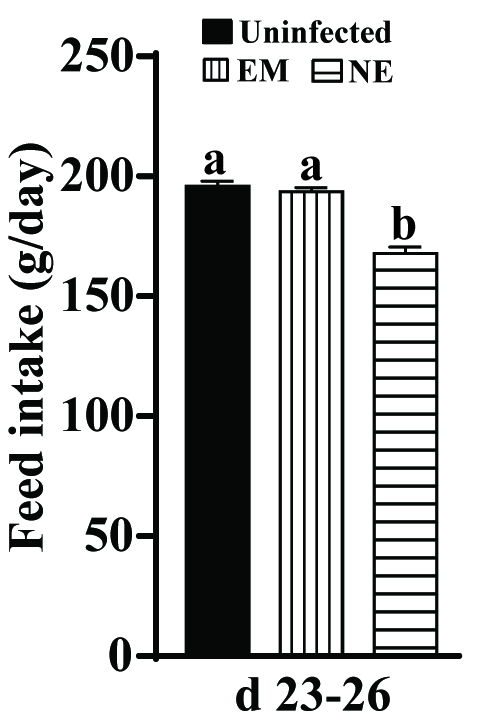

Supplement: Supplementary file 1 — Additional file 1: Supplement Figure 1. Subclinical necrotic enteritis infection reduces feed intake during NE phase. Feed intake of d 23–26 was compared among uninfected, E. maxima and NE groups. The graph showed mean ± SEM. Results are representative of 3 independent experiments. [file 40104_2020_441_MOESM1_ESM.tif]
